# Liquid Biopsy in Solid Tumours: An Overview

**DOI:** 10.1111/cyt.13485

**Published:** 2025-04-11

**Authors:** Pasquale Pisapia, Antonino Iaccarino, Giancarlo Troncone, Umberto Malapelle

**Affiliations:** ^1^ Department of Public Health University of Naples Federico II Naples Italy; ^2^ Department of Neurosciences, Reproductive and Odontostomatological Sciences University of Naples Federico II Naples Italy

**Keywords:** ctDNA, liquid biopsy, molecular oncology, molecular pathology, NGS

## Abstract

The advent of personalised and precision medicine has radically modified the management and the clinical outcome of cancer patients. However, the expanding number of predictive, prognostic, and diagnostic biomarkers has raised the need for simple, noninvasive, quicker, but equally efficient tests for molecular profiling. In this complex scenario, the adoption of liquid biopsy, particularly circulating tumour DNA (ctDNA), has been a real godsend for many cancer patients who would otherwise have been denied the benefits of targeted treatments. Undeniably, ctDNA analysis has several advantages over conventional tissue‐based analysis. One advantage is that it can guide treatment decision making, especially when tissue samples are scarce or totally unavailable. Indeed, a simple blood test can inform clinicians on patients' response or resistance to targeted therapies, help them monitor minimal residual disease (MRD) after surgical resections, and facilitate them with early cancer detection and interception. Finally, an equally important advantage is that ctDNA analysis can help decipher temporal and spatial tumour heterogeneity, a mechanism highly responsible for therapeutic resistance. In this review, we gathered and analysed current evidence on the clinical usefulness of ctDNA analysis in solid tumours.

## Graphical abstract

Potential applications of ctDNA analyses during the course of cancer patient journey. This figure was published as Figure 1 in Lung Cancer, Volume 172, Malapelle U, Pisapia P, Pepe F, Russo G, Buono M, Russo A, Gomez J, Khorshid O, Mack PC, Rolfo C, Troncone G., The evolving role of liquid biopsy in lung cancer, Pages 53–64, Copyright Elsevier (2022).

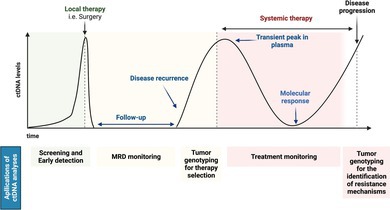

## 1 Introduction

The advent of personalised and precision medicine is rapidly shifting the paradigm of cancer treatment, thus improving the overall clinical outcomes of patients [1]. Tissue samples remain the “gold standard” input material to obtain tumour‐derived extracted nucleic acids [2]. However, tissue specimens, including histological and cytological specimens, are not always qualitatively and quantitatively sufficient for molecular testing. This is often either because some patients cannot undergo tissue biopsy owing to poor general conditions or to unreachable tumours, or because the material is inadequate for morpho‐molecular purposes [3, 4]. In this complex scenario, liquid biopsy has been a great game changer for cancer care. Indeed, since its adoption, some of the predictive and prognostic challenges pertaining to advanced stage cancer patients have been partially overcome, thereby increasing the number of patients eligible for targeted therapies [5, 6].

Liquid biopsy, an umbrella term that encompasses various body fluids, allows for minimally invasive assessments of the molecular status of solid tumours [6, 7, 8, 9, 10]. Generally, liquid biopsy samples contain a number of different analytes that, once isolated, can provide a thorough snapshot of tumour characteristics. Among the different types of analytes used in liquid biopsy, circulating tumour DNA (cfDNA), albeit representing only a small fraction of the total tumour‐derived circulating cell‐free DNA (cfDNA), is the most commonly adopted type [11, 12]. Currently, ctDNA has broad clinical application as a predictive, prognostic, preventive, and diagnostic marker. For instance, ctDNA analysis is widely adopted to guide clinicians through the treatment decision‐making process, especially when tissue samples are unavailable or when monitoring of treatment response is necessary. Moreover, it can be exploited for monitoring minimal residual disease after surgical resections and for improving early cancer detection and interception [13, 14]. Finally, as opposed to conventional tissue‐based analysis, ctDNA analysis is useful to detect temporal and spatial heterogeneity, a huge clinical challenge in cancer management to this day [15]. Unsurprisingly, ctDNA analysis has been rightfully adopted in various clinical trials [10, 16].

Given the current relevance and potential clinical impact of ctDNA on the future of cancer treatment, we have here reviewed and analysed the clinical usefulness of ctDNA analysis in solid tumours.

## 2 Cancer Screening, Early Detection and Prognosis

What makes ctDNA a potential revolutionary tool for cancer management is the concept that with a simple blood draw clinicians can trace temporal and spatial heterogeneity of different types of solid tumours, thereby overcoming some of the main constraints of conventional tissue biopsies. Indeed, thanks to the availability of highly sensitive and specific molecular technologies, ctDNA analysis is gaining momentum especially in cancer screening and early detection [13, 14]. Different assays are now available for these purposes. Among the many high throughput assays developed for cancer screening is the CancerSEEK assay. When applied to ctDNA samples, this assay is able to detect 16 gene mutations and proteins associated with eight common cancer types, namely, ovary, liver, stomach, pancreas, oesophagus, colo‐rectum, lung, and breast. In a validation study, CancerSEEK displayed a high sensitivity, ranging from 69% to 98% sensitivity, depending on the cancer type, and a specificity as high as 99% [17]. Another equally efficient assay developed for early cancer detection is targeted error correction sequencing (TEC‐Seq). This custom capture and sequencing approach allows sensitive and specific detection of low abundance sequence alterations using next generation sequencing (NGS). In a validation study, this approach correctly identifies 62% of stages I‐II of four cancer types, namely, colo‐rectum, ovary, breast and lung [18]. The high sensitivity and specificity of ctDNA analysis has also been evidenced by the Circulating Cell‐free Genome Atlas (CCGA) Consortium Study. Notably, this study highlights that targeted ctDNA methylation analysis can detect and localise numerous cancer types at varying stages [19, 20]. Still, similar lines of research have also amply demonstrated that increasing levels of ctDNA in the bloodstream [21, 22, 23, 24, 25, 26] and ctDNA methylation [27] are both associated with the worst prognosis across different cancer types. Overall, the above evidence demonstrates that the use of ctDNA analysis in detecting cancer in the very early stages has great clinical implications for many patients as it can spare them the need to undergo invasive procedures and unnecessary emotional stress.

## 3 Minimal Residual Disease

Besides being highly useful for early cancer detection, ctDNA analysis can also be used to monitor minimal residual disease (MRD) after surgery. Indeed, being noninvasive, it can be administered to patients frequently over time without adding additional physical postoperative discomfort. In this context, ctDNA results can update clinicians on the evolution of the disease after surgery, thereby helping them identify patients at a higher risk of recurrence and intervene accordingly [8]. Similarly, evidence in a milestone study by Diehl et al. highlights that the postoperative presence of high concentrations of ctDNA in the bloodstream of colorectal cancer patients is a reliable indicator of tumour evolution and relapse [28]. In another experience on breast cancer, postoperative detection of *PIK3CA* mutations in ctDNA analysis was associated with a high risk of relapse in early stage breast cancer patients [29]. Similar results have been reported in non‐small cell lung cancer (NSCLC) patients. Indeed, the seminal TRACERx study shows that the presence of mutations in ctDNA correlates with disease recurrence in stages I–III NSCLC [30]. Analogous findings were highlighted by Abbosh et al. In their study, the Authors reported that almost all early stage NSCLC patients with detectable ctDNA after surgical excision experienced disease recurrence [31].

Notably, ctDNA analysis is also useful to evaluate MRD in advanced stage cancer patients treated with systemic approaches. Indeed, in naïve metastatic colorectal cancer (mCRC) patients, Tie et al. demonstrated that early changes in ctDNA concentration after first‐line chemotherapeutic treatment may predict a subsequent radiological tumour response, suggesting that serial ctDNA measurement may complement the RECIST criteria for the management of cancer patients [32]. Consistently, another study reports that changes in ctDNA show a great dynamic range and correlation with changes in tumour burden in metastatic breast cancer patients receiving systemic therapies [33]. In advanced stage NSCLC patients, ctDNA levels have also been correlated with tumour burden [34]. Finally, ctDNA analysis has been shown to be a valid tool to predict treatment outcomes in patients undergoing targeted therapy [35].

Hence, postoperative analysis of the dynamic changes in ctDNA may be a valuable alternative to conventional tissue‐based analysis not only to monitor disease progression but also to predict treatment response in patients while avoiding the need for repetitive invasive procedures.

## 4 Target Treatments Administration in Advanced Stages

Besides being a useful tool for early stage screening, for tracking cancer evolution after surgery, and for evaluating the risk of relapse over time, ctDNA analysis is also very useful to support individualised treatment choices in advanced stage cancer patients. The reason is that in advanced stage cancer patients, tissue samples are either unavailable or too scarce to yield reliable results. Consequently, up to 43% of patients with advanced stage NSCLC do not receive adequate treatment [5]. On the other hand, our long‐lasting referral laboratory experience in molecular diagnostics has demonstrated the feasibility of ctDNA analysis in treatment‐naïve advanced NSCLC patients with inadequate tissue for *EGFR*, *KRAS* and *BRAF* mutation detection [36, 37, 38]. Encouraging results on the feasibility of exploiting ctDNA analysis to detect additional clinically relevant oncogenes in advanced stage NSCLC patients have also been reported in similar lines of research [39, 40, 41, 42, 43, 44]. A high tumour mutational burden in blood is currently being explored as a predictor of patients' response to immune‐checkpoint inhibitors [45]. Still, studies on mCRC patients have demonstrated that ctDNA analysis can successfully guide anti‐EGFR rechallenge therapy with panitumumab [46]. Finally, in advanced breast cancer, the SOLAR‐1 clinical trial—which compared the diagnostic accuracy of detecting *PIKA3CA* mutational status with ctDNA analysis with that of conventional tissue‐based analysis—showed high levels of specificity (97%) but low levels of sensitivity (55%) of *PIK3CA* testing in ctDNA [47]. This study thus suggests that although ctDNA may be a promising way to detect actionable mutations in highly heterogeneous advanced stage cancer types, improvements in detection efficiency are necessary to avoid the risk of obtaining false negative and false positive results, all to the detriment of patients.

## 5 Identification of Resistance Mechanisms

ctDNA can also be adopted to monitor clonal evolution and to identify resistance mechanisms to systemic treatment administration [48]. For example, in advanced stage NSCLC patients, ctDNA has been successfully used to evaluate the development of *EGFR* exon 20 p.T790M resistance point mutation after treatment with first‐ or second‐generation EGFR tyrosine kinase inhibitors (TKIs) [49]. In a similar way, in mCRC, ctDNA has been useful to monitor the development of *KRAS* resistance mechanisms in patients while undergoing anti‐EGFR treatment [50, 51]. Remarkably, in some cases ctDNA may have higher sensitivity than tissue samples in detecting resistant mechanisms and is therefore highly recommended for cancer patients who are more prone to tumour recurrence and progression than others. A case in point is oestrogen receptor (ER)‐positive, human epidermal growth factor receptor 2 (HER2)‐negative breast cancer patients. Indeed, in these patients, generally treated with endocrine therapy (with or without CDK4/6 inhibitor), ctDNA analysis of Oestrogen Receptor 1 (*ESR1*) gene mutations is strongly recommended in routine clinical practice [52, 53].

## 6 Pros and Cons of ctDNA Adoption

In a previous section, we mentioned that before ctDNA analysis can be fully adopted in routine clinical practice, several limitations should be taken into account. One limitation is that the clinical utility of ctDNA analysis is strongly influenced by the efficiency of the clinical context in providing complementary ancillary techniques capable of facilitating detection and interpretation of ctDNA results. For example, since ctDNA levels in the bloodstream are strongly affected by the clinical stage and disease burden [54], highly sensitive molecular technologies are required for the analysis [55]. The problem is that many healthcare facilities are unable to provide such advanced technologies. Another limitation that may prevent the full implementation of ctDNA in everyday clinical practice regards pre‐analytical issues. Among these issues are sample handling and storage. Indeed, owing to the very short half‐life of ctDNA, collection, extraction and storage strategies should be carefully optimised [56, 57]. Another limitation is the presence of clonal haematopoiesis of indeterminate potential (CHIP) in blood samples. Indeed, these haematopoietic mutations may be mistaken for solid tumour mutations, thereby giving rise to false positive results [58]. A final limitation is the lack of a standardised reporting system. Thus, efforts should be made to establish standardised and easily interpretable reporting of ctDNA results to ensure that patients receive the most effective treatment option based on the molecular profiles of their tumours [59].

An undoubted advantage of ctDNA with respect to traditional tissue biopsies is represented by the minimally invasive nature of the sampling procedures. Thus, it can be performed multiple times and sequentially, enabling the monitoring of the disease and changes developing under treatment pressure. In addition, ctDNA analysis may help to overcome intra‐ and inter‐tumour molecular heterogeneity, in particular in the case of progression of the disease and development of resistance mechanisms [60].

## 7 Daily Practice Adoption of ctDNA in Solid Tumours

The implementations of ctDNA have significantly modified the management of solid tumour patients. In advanced stage NSCLC patients, ctDNA analysis obtained the first clinical approval for EGFR TKI administration in patients naïve to any treatment (basal setting) without tissue availability or with an inadequate molecular result on tissue specimens, and for the identification of *EGFR* exon 20 p.T790M resistance point mutation in previously treated patients (resistance setting) [12]. Regarding *EGFR* testing, the first two platforms that obtained FDA and EMA approval were the real‐time polymerase chain reaction (RT‐qPCR) based cobas EGFR mutation test v2 (Roche, Basel, Switzerland) and TheraScreen EGFR RGQ PCR Kit (Qiagen, Hilden, Germany), followed by next generation sequencing (NGS) Guardant360 (Guardant Health, Palo Alto, CA, USA) and FoundationOne Liquid CDx (Roche) [61]. These latter were further approved for the detection of other clinically relevant targetable genomic alterations within advanced stage NSCLC patients [61, 62].

Regarding breast cancer, as discussed previously, ctDNA can be usefully adopted to evaluate *PIK3CA* gene mutational status. The results of the SOLAR‐1 trial led to the FDA approval of the companion diagnostic test Therascreen PIK3CA RGQ PCR Kit for the detection of *PIK3CA* mutations on both tissue and liquid biopsy specimens [47, 63]. Another important field of adoption of ctDNA analysis in breast cancer patients is for the identification of *ESR1* resistance mutations in ER‐positive, HER2‐negative breast cancer patients after endocrine therapy, with a digital droplet PCR (ddPCR) approach. In these patients, *ESR1* mutations are almost absent in treatment‐naïve primary tumours, whereas they are enriched in the metastatic setting under treatment pressure. Thus, there is a rationale to adopt liquid biopsy instead of invasive tissue sampling approaches [63].

Another setting in which ctDNA analysis obtained clinical approval was the early detection of CRC in asymptomatic average risk individuals undergoing screening through the identification of epigenetic changes, including DNA methylation of the septin9 (*SEPT9*) gene, with the RT‐qPCR approach namely EpiproColon test [64, 65]. In addition, as reported above, in the phase 2 CHRONOS trial, it has been demonstrated the potential of ctDNA analysis for guiding anti‐EGFR rechallenge therapy with panitumumab [46].

**FIGURE 1:**
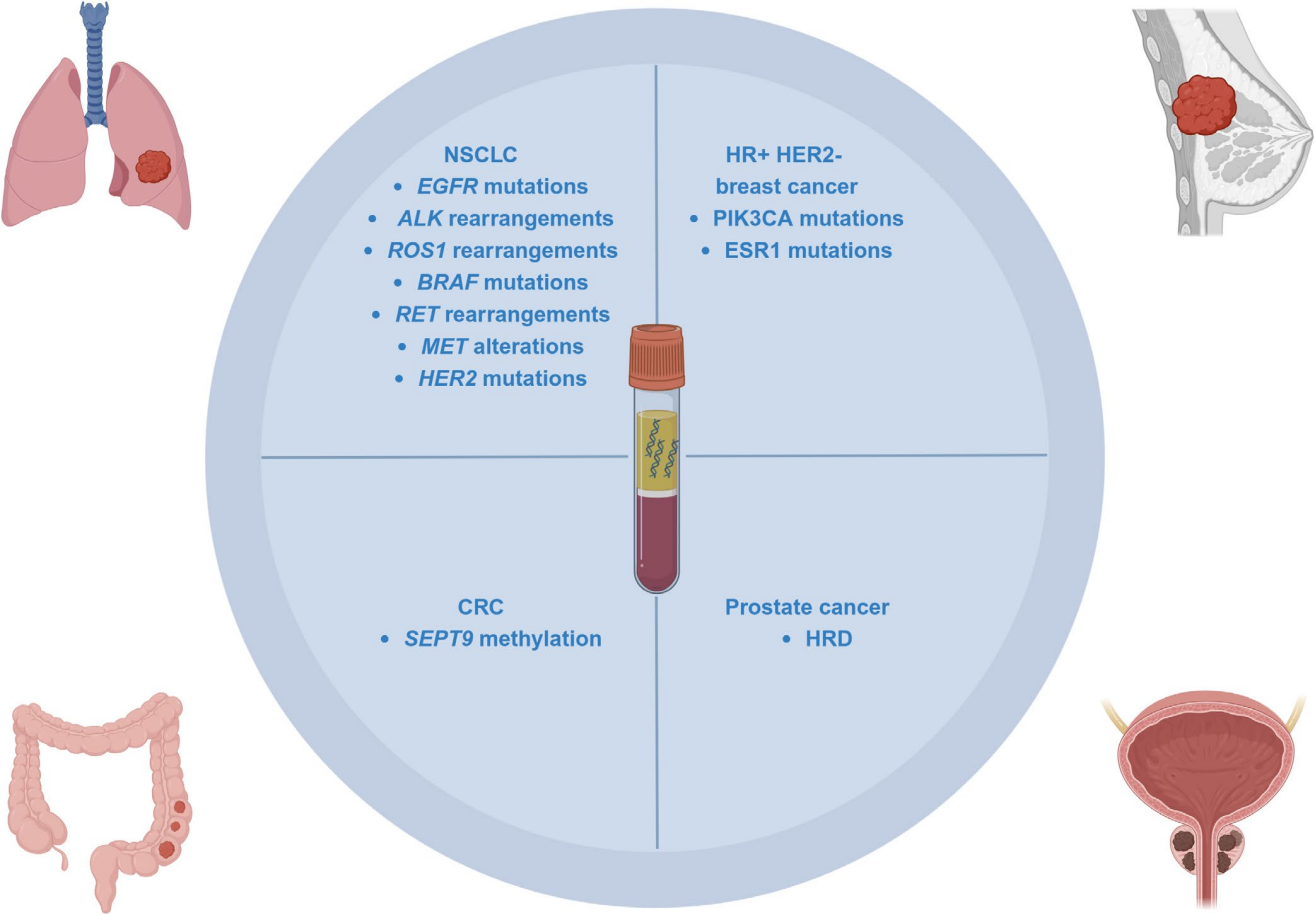
Daily practice adoption of ctDNA in solid tumours. This figure was created with BioRender (https://biorender.com) (accessed on 03 January 2025). *ALK*, Anaplastic Lymphoma Kinase; *BRAF*, V‐Raf Murine Sarcoma Viral Oncogene Homologue B1; CRC, colo‐rectal cancer; ctDNA, circulating tumour DNA; *EGFR*, Epidermal Growth Factor Receptor; *ESR1*, Oestrogen Receptor 1; *HER2*, Human Epidermal Growth Factor Receptor 2; HR, hormone receptor; HRD, homologous recombination deficiency; *KRAS*, Kirsten Rat Sarcoma Viral Oncogene Homologue; *MET*, MET Proto‐Oncogene, Receptor Tyrosine Kinase; NSCLC, non‐small cell lung cancer; *PIK3CA*, Phosphatidylinositol‐4,5‐Bisphosphate 3‐Kinase Catalytic Subunit Alpha; *ROS1*, ROS Proto‐Oncogene 1 Receptor Tyrosine Kinase; *RET*, REarranged during Transfection; *SEPT9*, septin9.

ctDNA analysis offers a minimally invasive approach for detecting homologous recombination deficiency (HRD) in advanced stage prostate cancer for the administration of poly(ADP‐ribose) polymerase inhibitors (PARPi). In particular, the FoundationOne Liquid CDx test (Roche) obtained FDA approval to guide the treatment of olaparib or rucaparib [66]. (Table 1 and Figure 1).

**TABLE 1:** Daily practice adoption of ctDNA in solid tumours.

Tumour type	Clinical adoption
NSCLC	*EGFR* mutations *ALK* rearrangements *ROS1* rearrangements *BRAF* mutations *RET* rearrangements *MET* alterations *HER2* mutations
HR+ HER2‐ breast cancer	*PIK3CA* mutations *ESR1* mutations
CRC	*SEPT9* methylation
Prostate cancer	HRD

Abbreviations: *ALK*, Anaplastic Lymphoma Kinase; *BRAF*, V‐Raf Murine Sarcoma Viral Oncogene Homologue B1; CRC, colo‐rectal cancer; ctDNA, circulating tumour DNA; *EGFR*, Epidermal Growth Factor Receptor; *ESR1*, Oestrogen Receptor 1; *HER2*, Human Epidermal Growth Factor Receptor 2; HR, hormone receptor; HRD, homologous recombination deficiency; *KRAS*, Kirsten Rat Sarcoma Viral Oncogene Homologue; *MET*, MET Proto‐Oncogene Receptor Tyrosine Kinase; NSCLC, non‐small cell lung cancer; *PIK3CA*, Phosphatidylinositol‐4,5‐Bisphosphate 3‐Kinase Catalytic Subunit Alpha; *RET*, REarranged during Transfection; *ROS1*, ROS Proto‐Oncogene 1 Receptor Tyrosine Kinase; *SEPT9*, septin9.

## 8 Conclusion and Future Perspectives

In conclusion, ctDNA analysis is an important arrow in the quiver of molecular pathologists and oncologists for the management of cancer patients. Indeed, this quick, simple, cost‐effective, and minimally invasive procedure may serve not only as a screening tool for detecting cancer in the early stages of the disease, but also as a valid prognostic and predictive tool for tracking cancer progression and treatment response over time. Despite holding great promise for the management of solid tumours, ctDNA does present some issues that need to be addressed before it can be fully implemented in everyday clinical practice. Indeed, pre‐analytical, analytical and post‐analytical issues should be dealt with to improve the sensitivity and specificity of this approach. Moreover, a standardised reporting system should be established to facilitate the interpretation and reporting of ctDNA results. In spite of these shortcomings, this line of research is definitely going to expand beyond ctDNA. Indeed, given the increasing popularity of ctDNA analysis, another fruitful field of investigation is represented by other body fluids. In fact, the concept of liquid biopsy can be extended to other body fluids, including cerebrospinal fluid (CSF), pleural effusion (PE), lymph, saliva and urine [9, 67]. In particular, these alternative sources of tumour nucleic acids from fluids that are more closely related to the metastatic site showed a higher sensitivity than blood in the detection of clinically relevant alterations for targeted treatments [68]. An important point that should be investigated is related to the modality of adoption of ctDNA into clinical practice. International guidelines suggested different approaches. In particular, a central point of discussion is the question of the adoption of a “plasma first” versus “tissue first” approach. Overall, a “third way” represented by a “complementary approach” can help us out of the impasse. In this scenario, if possible, concurrent tissue‐ and ctDNA‐based analysis should be performed on each patient to overcome the limitations and sum all the advantages of both approaches [56]. Another important issue is related to potential elevated costs associated with NGS. However, in two different experiences, it has been highlighted that the adoption of NGS allows saving personnel time dedicated to testing activities and to reduce the overall cost of testing per patient with respect to single gene approaches [69, 70]. In addition, it is already foreseeable that scientists will continue to investigate other sources of tumoral nucleic acids (e.g., other body fluids) [9], as well as other analytes [71].

## Author Contributions

All Authors conceived the review, collected the literature data, wrote the original draft, and approved the final version of the manuscript.

## Conflicts of Interest

Giancarlo Troncone reports personal fees (as speaker bureau or advisor) from Roche, MSD, Pfizer and Bayer for work unrelated to the current work. Umberto Malapelle has received personal fees (as consultant and/or speaker bureau) from Boehringer Ingelheim, Roche, MSD, Amgen, Thermo Fisher Scientific, Eli Lilly, Diaceutics, GSK, Merck and AstraZeneca, Janssen, Diatech, Novartis, Hedera, and Menarini for work performed unrelated to the current work. The other authors declare no potential conflicts of interest.

## Data Availability

The authors have nothing to report.
